# P04.76. Characteristics of yoga practice in an undergraduate student sample

**DOI:** 10.1186/1472-6882-12-S1-P346

**Published:** 2012-06-12

**Authors:** K Riley, C Park, M Marks, T Braun

**Affiliations:** 1 University of Connecticut, Storrs, USA; 2 Kripalu Center for Yoga and Health, Stockbridge, USA

## Purpose

Young adults are increasingly practicing yoga (Barnes et al., 2008) and yoga interventions have been shown to decrease stress and anxiety. However, little is known about the characteristics of young adults who engage in yoga or the correlates of yoga practice outside of clinical contexts. Our objective was to characterize students who practice yoga and to examine correlates of recent yoga practice.

## Methods

As part of participant pool screening, an online survey assessed a group of undergraduate students.

## Results

Three hundred forty-two students (53.6%) reported ever having done yoga and 296 students (46.4%) reported never having done yoga. Our sample of yoga users was similar to national samples: 61% Christian, 75.2% White, 8.1% Hispanic, and 77.7% female. Students who had practiced yoga reported more spirituality (p<.02) and rated their health as more important (p<.003) than those who had never used yoga. Additionally, those who had used yoga were more likely female (p<.001) and less likely Hispanic (13.5% vs. 8.1% ). Groups did not differ in age, economic status, SAT scores, being born in the US, English as first language, or race. Among those who had ever used yoga, 110 (32.3%) had attended a class recently (in the past three months). Similar to previous studies, gender (p<.05), other exercise (p<.05), and motivation to be healthy (p<.001) correlated with having practiced yoga recently, but these variables were unrelated to the number of classes recently attended. Being born outside of the US (p<.01) and not having English as one’s first language (p<.05) were correlated with more yoga classes attended recently.

## Conclusion

University students who practice yoga are similar to nationally representative adult samples, but in much higher proportion. Yoga appears to be part of a healthy lifestyle for undergraduates. Future research is needed to understand causal relations and extent to which students’ yoga practice changes throughout the college years.

